# The substrate specificities of sunflower and soybean phospholipases D using transphosphatidylation reaction

**DOI:** 10.1186/1476-511X-10-196

**Published:** 2011-11-01

**Authors:** Slim Abdelkafi, Abdelkarim Abousalham

**Affiliations:** 1 Organization and Dynamics of Biological Membranes, UMR 5246 ICBMS, CNRS-Université Claude Bernard Lyon 1, Bâtiment Raulin, 43, boulevard du 11 novembre 1918, 69622 Villeurbanne, Cedex, France; 2 Université de Sfax, Centre de Biotechnologie de Sfax, Laboratoire des Bio-Procédés Environnementaux, Sfax, Tunisia

## Abstract

**Background:**

Phospholipase D (PLD) belongs to a lipolytic enzyme subclass which catalyzes the hydrolysis and transesterification of glycerophospholipids at the terminal phosphodiester bond.

**Results:**

In this work, we have studied the substrate specificity of PLDs from germinating sunflower seeds and cultured-soybean cells, using their capacity of transphosphatidylation. In the presence of a nucleophilic acceptor, such as [^14^C]ethanol, PLD catalyzes the production of phosphatidyl-[^14^C]-ethanol. The resulting product is easily identified since it is well separated from the other lipids by thin-layer chromatography. The main advantage of this assay is that the phospholipid used as substrate does not need to be radiolabelled and thus allow us a large choice of polar heads and fatty acids. *In vitro*, we observed that sunflower and soybean cell PLD show the following decreasing order of specificity: phosphatidylcholine, phosphatidylethanolamine and phosphatidylglycerol; while phosphatidylserine and phosphatidylinositol are utilized much less efficiently.

**Conclusions:**

The substrate specificity is modulated by the fatty acid composition of the phosphatidylcholine used as well as by the presence of other charged phospholipids.

## Background

Phospholipase D (PLD) (phosphatidylcholine phosphatidohydrolase, [EC3.1.4.4]) is a ubiquitous enzyme present in mammals, plants and bacteria [1-3]. PLD catalyses two reactions (i) hydrolysis of phospholipids to produce phosphatidic acid (PA) and a free polar head group such as choline in the case of phosphatidylcholine (PC), and (ii) transphosphatidylation reaction which, in the presence of a primary alcohol, PLD leads to the formation of the corresponding phosphatidyl alcohol [3]. The latter product is less subject than PA to further metabolism, and can easily be separated from the substrate and other hydrolytic products by performing thin-layer chromatography (TLC). Transphosphatidylation is a useful reaction synthesizing natural phospholipids, such as phosphatidylserine (PS) and phosphatidylglycerol (PG), and novel artificial phospholipids [4]. These phospholipids have been used for pharmaceuticals, foods, cosmetics, and other industries. Transphosphatidylation is usually carried out in a biphasic system consisting of water and water-insoluble organic solvents. The reaction is usually accompanied with various amounts of the hydrolysis product PA [5,6].

PLD was first identified in plants where an isoform called PLDα is widespread. The plant PLDα has been purified for the first time to homogeneity in 1993 in two different laboratories from the cabbage leaves [7] and castor bean endosperm [8]. Twelve PLD isoforms grouped into five types (PLDα, PLDβ, PLDγ, PLDδ and PLDζ) could be identified in *Arabidopsis thaliana *[9]. Subsequently, it has been described in mammals, bacteria and yeasts. PLD activity and PA levels increase rapidly in plant tissues under various stress conditions [5]. Increasing studies indicate that PLD and PA are involved in regulating plant growth, development, and response to environmental and biotic stresses [5,10-12]. The function of PLD and PA has been linked to the survival, proliferation, and reproduction of cells and organisms. Latest results indicate that specific PLD- and PA-mediated signalling play important roles in plant biomass production and response to water deficits and nutrient deficiency [13,14].

PLDs from different sources share some characteristics in their molecular organization and constitute, together with some other evolutionary related proteins, the PLD superfamily [15]. Mammalian PLD activities have been found to preferentially catalyze platelet-activating factor and PC phosphatidylethanolamine (PE), phosphatidylinositol (PI), and PI-glycan [16-18].

Work from our laboratory have examined previously the fatty acid specificity of PLD purified from germinating sunflower seeds using wide range of PC compounds with various fatty acid contents [19]. We have shown that sunflower PLD is most active on medium-chain fatty PC compounds. In general, PLDs hydrolyses a broad range of phospholipids with different head groups including PC, PE, PG, PS, PI, lyso-PC, cardiolipin, and plasmalogens with preferences depending on the enzyme source and isoform [19]. Despite these informations, the study of substrate specificity of pure PLD using the transphosphatidylation reaction is limited. In order to systematically investigate the substrate specificity of purified PLD from germinating sunflower seeds and cultured-soybean cell, we used the transphosphatidylation reaction, with [^14^C]-ethanol as the acceptor. The resulting phosphatidyl-[^14^C]-ethanol could be easily identified by performing TLC, since it was clearly separated from the other compounds. During this reaction, the various phospholipids used do not need to be radiolabelled, so that a wide choice of possible phospholipids is available.

## Materials and methods

### Purification of sunflower and soybean PLDs

Acetone sunflower seeds powder was prepared from germinating sunflower seeds using the standards procedures developed at our laboratory [19-21]. The delipidated powder was extracted with 50 mM Tris/HCI, pH 7.5, and the purification was carried out using the procedure developed by Abousalham et al. [7] for use with the cabbage enzyme. Soybean PLD was purified from Soybean (*Glycine max *L.) suspension-culture cells as described previously by Abousalham et al. [22]. Pure PLDs were aliquoted and stored at - 80°C until use.

### Protein quantification and gel electrophoresis

Protein concentrations were determined routinely using the Bradford procedure [23] with Bio-Rad Dye Reagent and bovine serum albumin as the standard. Samples were separated by 12% sodium dodecyl sulfate (SDS) polyacrylamide gel (PAGE) as described by Laemmli [24]. The apparent molecular masses of proteins were estimated by co-electrophoresis of marker proteins (Biorad, Hercules, CA, USA) with masses ranging from 14.4 to 116 kDa. The protein in sample buffer (0.9 g glycerol, 0.1 ml 1% bromo-phenol blue, 1 mL 10% (w/v) SDS, and 0.1 mL mercaptoethanol) was heated for 5 min in boiling water and applied to the gel. The proteins separated on the SDS-PAGE were stained with Coomassie Brilliant Blue R-250.

### PLD assay

PLD activity was assayed spectrophotometrically by measuring the free choline released upon PC hydrolysis, using a continuous method [19] adapted for microplates (96 wells) from Takamara and Taylor [25]. Choline was continuously transformed into betain by means of choline oxidase, which simultaneously yielded H_2_O_2_. In the presence of 4-aminoantipyrine and sodium 2-hydroxy-3, 5-dichlorobenzenesulfonate, the H_2_O_2 _was used instantaneously by the added peroxidase to form a colored product absorbing light at 500 nm. Optical density measurements were performed using a microplate scanning spectrophotometer (PowerWave, Bio-Tek instruments). The egg PC substrate was prepared by dispersing egg PC in an equimolar mixture (0.83 mM) of SDS and Triton X-100. The assay mixture contained 50 mM Tris/HCl, pH 8.0, 20 mM CaCl_2_, 1.7 mM 4-aminoantipyrine, 9 mM sodium 2-hydroxy-3, 5-dichlorobenzenesulfonate, 0.5 U choline oxidase and 0.5 U peroxidase. The reaction was initiated by adding PLD and the substrate (egg PC mixed micelles, 0.26 mM, final concentration) and absorbance measurements were carried out every 30 s for 5 min. The amounts of free choline released were quantified, based on a standard curve obtained with pure choline. One unit of PLD activity was defined as the amount of enzyme releasing 1 μmol of choline/min under the experimental conditions specified above.

### TLC assay for transphosphatidylation activity

The reaction mixture (0.5 mL final volume) was composed of 0.26 mM phospholipid dispersed in an equimolar mixture of SDS and Triton X-100, 50 mM Tris-HCl (pH 8), 20 mM CaCl_2 _and 2% ethanol (final concentration). After incubating this mixture with the appropriate amount of enzyme for 10 min at 37°C, the phospholipids were extracted with 0.8 mL of chloroform-methanol (2:1, v/v) under vigorous shaking. Phase separation was facilitated by centrifugation for 10 min at 2, 000 rpm. The lower organic phase was collected and transferred to a 5-mL test tube, where it was dried over anhydrous magnesium sulfate (MgSO_4_). Once MgSO_4 _had precipitated, 0.4 ml of the clear organic phase was transferred to a 2-ml vial with a screw-cap and dried under a stream of nitrogen. The samples were dissolved in 40 μL of chloroform methanol (2:1) and applied along with PEt standard to aluminum TLC plates. Plates were developed in a solvent system consisting of chloroform/methanol/acetic acid (65:25:4 v/v/v). In this solvent system, PEt (Rf = 0.54) migrates faster than PA (Rf = 0.3) and the other phospholipid substrates (Rf = 0-0.2). The phospholipids were revealed by iodine vapour, and the radioactive spots corresponding to PEt were cut out from the plates and the radioactivity was quantitated by liquid scintillation count in a Beckman LS 5000 TD beta counter after the disappearance of the iodine vapour [26].

## Results and discussion

**Figure 1:**
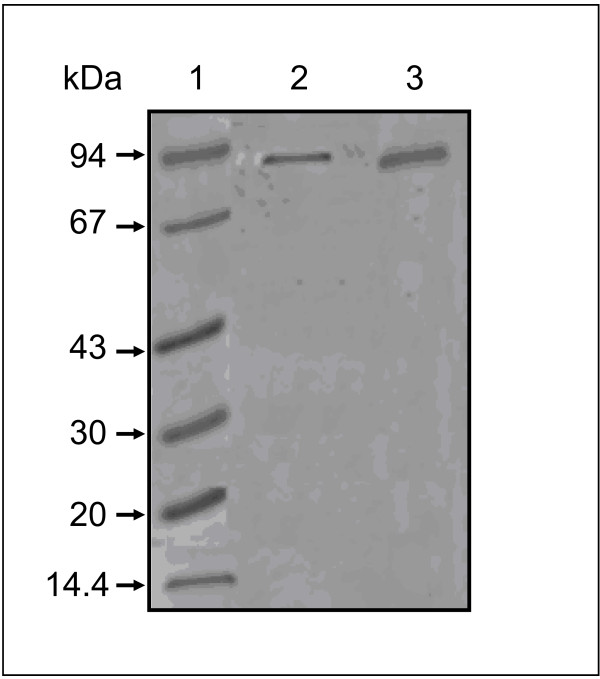
**SDS-PAGE of purified sunflower and soybean PLDs**. The gel was stained with Coomassie brilliant blue to reveal the proteins. Lane 1, molecular mass markers; lane 2, pooled fractions containing active sunflower PLD; lane 3, pooled fractions containing active soybean PLD.

It has been reported that most plant PLDα was strongly bound to a hydrophobic support in the presence of Ca^2+ ^ions [7]. We used this property to purify PLDs collected from germinating sunflower seeds and cultured-soybean cells. The purification was performed using an Octyl-Sepharose CL-4B column. The PLD activities were eluted by chelating the Ca^2+ ^ions with EDTA. Highly purified PLD from sunflower and soybean devoid of any detectable contaminants, give a single protein band at a position corresponding to a molecular mass of around 90-92 kD for each PLD (Figure 1). These molecular masses are similar to the values reported in literature [8,19,22,27]. PLD from germinating sunflower seeds and cultured-soybean cell possess both hydrolytic and transphosphatidylation activities which are both dependent upon the presence of Ca^2+ ^ions. The optimal pH for PLD activity is 7.5, using 10 mM CaCl_2_. No activity was detected in the absence of Ca^2+ ^ions. Time course experiments showed that the reaction progressed linearly for at least 30 min (data not shown).

**Figure 2:**
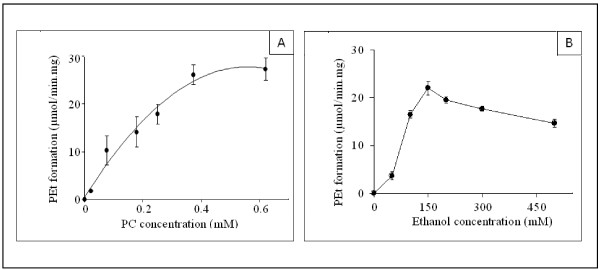
**The concentration-dependency of sunflower PLD activity**. (A) Dependency of sunflower PLD specific activities upon PC concentrations. PEt was determined as described in Materials and Methods. Each assay (0.5 ml total volume) was carried out with 5 μg of sunflower PLD and 150 mM ethanol (final concentration) in the presence of 0.5 μCi of [^14^C]ethanol. Values are means ± SD (Standard deviation) from three experiments. (B) Effects of the ethanol concentrations on the specific activities of PLD. PEt was determined as described in Materials and Methods. Each assay (0.5 ml total volume) was carried out with 5 μg of sunflower PLD and 0.5 mM (final concentration) PC vesicle suspensions. Results are shown as means from two independent experiments.

The concentration-dependency of sunflower PLD activity, using soybean PC as substrate, is shown in Figure 2A. The PEt formation increased hyperbolically with the PC concentration. The kinetic constants for phospholipids were determined by varying the concentrations of substrate, in range of 0-0.6 mM (Figure 2A). The apparent Km and Vmax values estimated were 0.2 mM and 27 μmol/min.mg respectively. Similar results were obtained using soybean cell PLD (data not shown). Savoy cabbage PLD has been reported to show a similar type of kinetic behaviour [28]. The amount of PEt formed increased linearly with increasing amounts of PLD up to 25-30 μg of protein at 0.5 mM soybean PC (data not shown). The effect of varying the ethanol concentration on PEt formation was examined and the results are presented in Figure 2B. The maximum PEt formation was produced at 150 mM ethanol, and a slight inhibition occurred at higher ethanol concentrations. These results are qualitatively in agreement with previous observations on rat brain synaptosomal PLD [4,29]. During the transphosphatidylation reaction, ethanol and water act as competitive nucleophiles. It is worth noting however that the water and ethanol concentrations were 55 M and 150 mM, respectively. This indicates that PLD has a strong preference for ethanol as a co-substrate. These data nevertheless suggest that the transphosphatidylation activity of PLD may play a more important role than hydrolysis in the turnover and synthesis of phospholipids.

**Figure 3:**
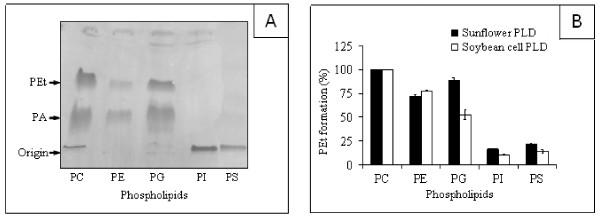
**Transphosphatidylation reaction products obtained using sunflower PLD**. (A) TLC pattern of PEt and PA formation. Incubation conditions and lipid chromatography are described in Materials and Methods. The arrows point to the PEt, PA, and the origin of application. (B) Substrate specificities of sunflower and soybean cell PLDs. The final concentration of all the phospholipids used was 0.5 mM. PLD was assayed (0.5 ml total volume) by measuring the PEt formation as described in Materials and Methods. Each assay was carried out with 5 μg of sunflower or soybean cell PLD in the presence of 150 mM ethanol (final concentration) containing 0.5 μCi of [^14^C]ethanol. Results are shown as means from two independent experiments.

The transphosphatidylation capacity of PLD was used to examine its activity on various commercially available classes of phospholipids in the form of sonicated vesicles at a constant final concentration of 0.5 mM. Chromatogram of the transphosphatidylation reaction products obtained using sunflower PLD is shown in Figure 3A. Using ethanol as an acceptor, the main phospholipid produced after 10 min was PEt (Rf = 0.54), as the result of the transphosphatidylation activity of PLD. PA (Rf = 0.3) was also formed as a side product of the hydrolytic activity. The zwiterionic PC molecule is the most effective substrate for sunflower and soybean PLD (Figure 3B). PE (71-77% of the maximal activity) and PG (89-52% of the maximal activity) are also used equivalently by both enzymes. We observed a relatively small but significant activity with PS (21-14% of the maximal activity) and PI (16-10% of the maximal activity). Plant PLD require Ca^2+ ^ions to express their maximal activity, and one should recall that these negatively charged phospholipids form strong complexes with calcium ions [19]. A similar preference for PC, the major phospholipid in eucaryotic membranes, was reported in numerous studies with crude or partially purified PLD (for a review, see [30]). A cytosolic PLD preferentially utilizing PE, PC, and PI was identified in various bovine tissues [10].

**Figure 4:**
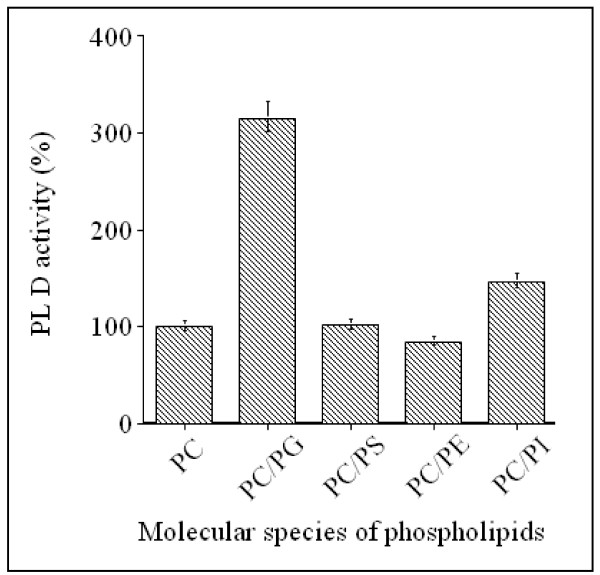
**Effect of other phospholipid classes on PC hydrolysis by PLD**. All the phospholipids were sonicated separately and mixed with preformed PC vesicles in the assay medium at a 1/1 molar ratio. Sunflower PLD activity was assayed by measuring water-soluble choline as described in Materials and Methods. The results are representative of three experiments.

In order to further examine the effects of charged phospholipids (PG, PS and PI) and zwiterionic PE on PC hydrolysis, sunflower PLD activity was evaluated by estimating the amounts of water-soluble choline released, using soybean PC as substrate. All the phospholipids used were sonicated separately and mixed with preformed PC vesicles in the assay medium at a 1/1 molar ratio. As shown in Figure 4, taking the rate of hydrolysis of soybean PC alone to be 100%, the presence of PG vesicles increased the PLD activity three fold and PI had weak stimulatory effect (145%) on PC hydrolysis, whereas, PS and PE had no significant effect. Similar results were obtained when PC and the other phospholipid species were co-sonicated and used as mixed substrates (data not shown). Under these conditions, the PG is the best substrate for sunflower PLD. The above results are probably underestimated, since the non choline containing phospholipids, which are potential substrates (see Figure 3), act as competitive inhibitors for the PC molecules. These data show the possibility that PC hydrolysis depends upon the presence of other lipids present in the assay medium.

**Figure 5:**
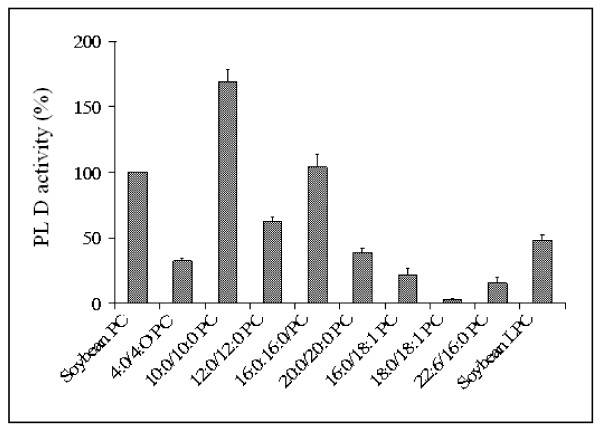
**Fatty acid specificity of PLD hydrolysis of various PC species**. The final concentration of each phospholipid used was 0.5 mM. Sunflower PLD activity was assayed by measuring water-soluble choline as described in Materials and Methods. Results are shown as means from two independent experiments.

We reported previously [19] that the activity of sunflower PLD on PC molecules depends significantly on the nature of the constituent fatty acyl chains. The hydrolytic activity decreased markedly when the acyl chain was longer than 10. In this study, the effects of the fatty acid composition on the hydrolysis of PC by soybean PLD were also investigated (Figure 5). For this purpose we used eight molecular species of PC containing various acyl chains, 4:0/4:0 PC, 10:0/10:0 PC, 12:0/12:0 PC, 16:0/16:0 PC, 20:0/20:0 PC, 16:0/18:1 PC, 18:0/18:1 PC, 22:6/16:0 PC, in the *sn*-1 and *sn*-2 positions, respectively. Taking the rate of hydrolysis of soybean PC to be 100%, the values at a breakdown level of 4:0/4:0 PC, 10:0/10:0 PC, 12:0/12:0 PC, 16:0/16:0 PC and 20:0/20:0 PC were 32%, 168%, 62%, 103% and 38%, respectively. The maximum enzyme activity was observed with PC molecules containing an acyl chain with 10 carbon atoms. Surprisingly the level of hydrolysis was very low with the following unsaturated species: 16:0/18:1 PC (21%), 18:0/18:1 PC (3%) and 22:6/16:0 PC (15%). Apparently, soybean PLD has a preference for saturated acyl chains of medium size (10:0 to 12:0) as well 16:0. Molecules of PC with acyl chains shorter than C6 or longer than C18 are poor substrates. The two most abundant fatty acids found in the *sn*-1 position of natural phospholipids are palmitic and stearic acids [31]. PC contains more palmitic than stearic acid, whereas the reverse occurs with the other phospholipid classes. Although PLD catalyzes reactions mainly involving the phospholipid polar head group, the present data clearly indicate that the specificity of plant PLD may also depend on the fatty acid composition of the PC species. These findings are compatible with previous data showing that an alkyl substitution at the PC *sn*-2 position affects its ability to act as a substrate for cabbage PLD [32]. As shown in Figure 5, soybean PLD also showed lysophospholipase D activity when water soluble soybean LPC was used as a substrate (50% of the maximal rate recorded with soybean PC). The two acyl chains are probably necessary for a satisfactory degree of catalytic efficiency to be achieved. These results are in agreement with those published previously. Shimbo et al. [33] have reported however that LPC was hydrolyzed about 50 times more slowly than PC by PLD from *Streptomyces antibioticus*.

One might have expected PLD, acting upon the polar heads of phospholipids, to be less dependent on the "quality" of the interface. Like many other lipolytic enzymes [34], PLD activity seems to depend however upon the physico-chemical properties characteristic of the lipid/water interface, in addition to the chemical nature of the phospholipid polar head group. In this context, research by Abousalham et al. [35], using phospholipid monomolecular films as a substrate, is of particular interest. These authors have shown that 12:0/12:0 PC are optimally hydrolyzed at a surface pressure of 10 mN/m. Another family of lipolytic enzymes, phospholipase C, also acting upon the polar heads of phospholipids, shows the same type of behavior. For instance Moreau et al. [36] have studied the hydrolysis of PC films by phospholipase C and the optimal surface pressure was around 15 mN/m, using 10:0/10:0 PC and 12:0/12:0 PC films as substrates. However, 10:0/10:0 PC was hydrolyzed at about twice the rate of 12:0/12:0 PC.

A detailed description of phospholipid specificity is necessary to be able to interpret the physiological action of PLD on natural membranes. Our results show clearly that the best substrate for plant PLD is the PC species and that the substrate specificity of these enzymes is also determined by the fatty acid composition and by the phospholipid environment.

## Competing interests

The authors declare that they have no competing interests.

## Authors' contributions

SA and AA carried out all the studies and analyzed the data. All authors have read and approved the final manuscript.
